# Pathogenic bacteria and treatment resistance in older cardiovascular disease patients with lung infection and risk prediction model

**DOI:** 10.1515/biol-2022-0756

**Published:** 2023-12-13

**Authors:** Hongbo Liu, Liyan Xie, Cong Xing

**Affiliations:** The Municipal Hospital of Qingdao Cadre Health Section, Qingdao, Shandong 266000, China; Qingdao Municipal Hospital, Health Care Clinic, Qingdao, Shandong 266000, China; Health Promotion Centre, Baoji Maternal and Child Health Care Hospital, Baoji, Shaanxi 721000, China

**Keywords:** cardiovascular disease, pulmonary infection, pathogenic bacteria, drug resistance, risk prediction model

## Abstract

This study analyzes the distribution of pathogenic bacteria and their antimicrobial susceptibilities in elderly patients with cardiovascular diseases to identify risk factors for pulmonary infections. A risk prediction model is established, aiming to serve as a clinical tool for early prevention and management of pulmonary infections in this vulnerable population. A total of 600 patients were categorized into infected and uninfected groups. Independent risk factors such as older age, diabetes history, hypoproteinemia, invasive procedures, high cardiac function grade, and a hospital stay of ≥10 days were identified through logistic regression. A predictive model was constructed, with a Hosmer–Lemeshow goodness of fit (*P* = 0.236) and an area under the receiver operating characteristic curve of 0.795, demonstrating good discriminative ability. The model had 63.40% sensitivity and 82.80% specificity, with a cut-off value of 0.13. Our findings indicate that the risk score model is valid for identifying high-risk groups for pulmonary infection among elderly cardiovascular patients. The study contributes to the early prevention and control of pulmonary infections, potentially reducing infection rates in this vulnerable population.

## 1 Introduction

The incidence of cardiovascular disorders has been escalating in recent years, a trend that is concomitant with both the aging demographic and the enhancement in living standards. In the geriatric population aged 60 years and above, approximately 10% exhibit clinical manifestations associated with cardiovascular maladies, posing a significant threat to patient morbidity and mortality [1,2]. Cardiovascular pathologies are predominantly linked to arteriosclerotic underpinnings; patients manifesting acute clinical presentations are predominantly classified as high-risk, while those with chronic conditions are more inclined toward a protracted course of the disease. This extended trajectory often culminates in attenuated immunological responses and an elevated susceptibility to pulmonary infections [3]. Pulmonary infection refers to the microbial invasion of the lower respiratory tract and pulmonary parenchyma. Particularly in the elderly, the physiological decline associated with aging manifests as diminishing immunological capabilities and a compromised capacity to fend off pathogenic onslaughts. Concurrently, age-related respiratory mucosal atrophy, aberrant ciliary motility, diminished respiratory function, and reduced lung compliance are ubiquitous phenomena. Under the influence of concomitant cardiovascular disorders, patients may exhibit various degrees of pulmonary congestion and edema, leading to alveolar reduction, diminished infection resistance, and resultant pulmonary infections. Moreover, clinical management of cardiovascular diseases frequently necessitates invasive interventions like intubation or mechanical ventilation, rendering the patient more susceptible to nosocomial pulmonary infections due to pathogenic exposure [4,5].

Overutilization of antibiotics has seen a troubling increase in recent years, precipitated by the rapid development of these therapeutic agents. This has further complicated the epidemiology and antimicrobial resistance patterns of pathogenic bacteria responsible for pulmonary infections [6]. Despite voluminous research endeavors in this domain, a reliable risk prediction model for pulmonary infections in geriatric patients with cardiovascular diseases remains elusive, thereby constraining its applicative utility in prognostic settings [7].

Against this backdrop, the present study endeavors to analyze the distribution of pathogenic bacteria and their antimicrobial susceptibilities in the context of pulmonary infections in the elderly population with cardiovascular diseases. By identifying the risk factors associated with such infections, we aim to establish a robust risk prediction model. This model intends to serve as a valuable clinical tool for the early prevention of pulmonary infections in this vulnerable population. Such predictive insights are conducive to the timely initiation of targeted preventive nursing interventions as well as the implementation of standardized diagnostic, therapeutic, and nursing protocols, as elucidated in the subsequent sections of this article.

## 2 Materials and methods

### 2.1 General data

From January 2017 to December 2019, 600 patients, 312 men and 288 females, aged 60–89, with an average age of 73.54 ± 8.76 years, were selected from our hospital’s patients with cardiovascular disease. Primary diseases included hypertension (238 cases), coronary heart disease (206 cases), pulmonary heart disease (51 cases), cardiomyopathy (48 cases), congenital heart disease (32 cases), and valvular heart disease (25 cases). Based on the pulmonary infection status, patients were divided into infected group (*n* = 71 cases) and uninfected group (*n* = 529 cases). Inclusion criteria were (1) patients with cardiovascular disease, (2) age ≥60 years, and (3) patients with complete clinical data. Exclusion criteria were (1) blood or immune system-related diseases, (2) liver and kidney insufficiency, and (3) malignant tumors. The criterion for pulmonary infection was pulmonary infection secondary to cardiovascular disease and occurred during hospitalization. The diagnosis of pulmonary infection was based on the “Diagnostic Criteria for Nosocomial Infection” [8]: (1) body temperature ≥38℃, (2) white blood cell count ≥10.0 × 10^9^/L, (3) lung auscultation showing rales of both lungs, (4) cough, expectoration, and other symptoms, (5) X-ray films showing inflammatory changes in the lungs, and (6) sputum culture showing the growth of pathogenic bacteria. The clinical data of 600 patients were collected through the hospital database, and the data were analyzed and processed by SPSS 26.0. The Hospital Theory Committee has approved the study.


**Informed consent:** Informed consent has been obtained from all individuals included in this study.
**Ethical approval:** The research related to human use has been complied with all the relevant national regulations, institutional policies and in accordance with the tenets of the Helsinki Declaration, and has been approved by the Hospital Theory Committee.

### 2.2 Methods

#### 2.2.1 General data collection

Patient baseline data were meticulously collated and comprised the following variables: (1) general information: gender, age, previous disease history, invasive operation, cardiac function grade, and hospital stay, (2) auxiliary examination involved blood routine examination, blood gas analysis, imaging diagnosis, biochemical indicators, etc.

#### 2.2.2 Detection of pathogenic bacteria

Prior to the diagnostic evaluation, patients were instructed to gargle multiple times with isotonic saline solution and expectorate deep respiratory tract sputum into a sterile receptacle. Pathogenic bacteria were then isolated from the expectorated sputum and cultured in accordance with the “National Clinical Laboratory Procedures” [9]. The centrifugally obtained sediment was inoculated onto sheep blood agar and MacConkey agar plates, and subsequently incubated at ambient temperature for 24 h. Pathogenic bacterial identification was performed utilizing the VITEK 2 COMPACT identification system in alignment with the protocols established by French BioMérieux. Quality control strains: *Escherichia coli* ATCC25922, *Staphylococcus aureus* ATCC25923, *Pseudomonas aeruginosa* ATCC27853, from the Institute of Biology, Chinese Academy of Sciences, and the drug susceptibility test was detected employing the K–B test method.

### 2.3 Statistical methods

Statistical analyses were performed using the SPSS version 20.0 software suite. The *χ* ^2^ test was employed to assess differences between the groups. Within the framework of this retrospective investigation, the primary outcome measure was identified as the incidence of pulmonary infections. Patients were divided into an infected group and an uninfected group according to whether pulmonary infection occurred, and the difference between the infected group and the uninfected group was analyzed. Risk variables predisposing older individuals with cardiovascular disease to subsequent pulmonary infections were scrutinized via logistic regression analysis, culminating in the development of a predictive regression model. The discriminative ability of the formulated model was subsequently assessed using receiver operating characteristic (ROC) curve analysis. A statistical significance threshold was established at *P* < 0.05 for all inferential analyses.

## 3 Results

### 3.1 Pathogenic bacterial distribution in elderly cardiovascular patients

A total of 93 microbial strains were isolated from the sputum samples of 71 patients. This microbial array comprised 22 Gram-positive strains, accounting for 23.66% of the total isolates, in conjunction with 67 Gram-negative strains, which constituted 72.04%, and four fungal isolates, representing 4.30%. A statistically significant predominance was observed in the proportion of Gram-negative strains relative to other microbial classifications (*P* < 0.05) (Table 1).

**Table 1:** Distribution of pathogenic bacteria in the samples from this study

Pathogenic bacteria	Number of strains (*n*)	Composition ratio (%)
**Gram-positive bacteria**		
*S. aureus*	14	15.05
*Streptococcus pneumoniae*	6	6.45
*Enterococcus faecium*	1	1.08
*Enterococcus faecalis*	1	1.08
**Gram-negative bacteria**		
*K. pneumoniae*	19	20.43
*E. coli*	16	17.20
*P. aeruginosa*	15	16.13
*Acinetobacter baumannii*	7	7.53
*Enterobacter cloacae*	6	6.45
*Stenotrophomonas maltophilia*	3	3.23
*Proteus mirabilis*	1	1.08
**Fungi**		
*Candida albicans*	2	2.15
*Candida tropicalis*	1	1.08
*Candida krusei*	1	1.08

### 3.2 Antibiotic sensitivity and resistance profiles in key bacterial strains

*Klebsiella pneumoniae*, *E. coli*, and *P. aeruginosa* were sensitive to imipenem, meropenem, and teicoplanin. Conversely, the highest rate of drug resistance was observed in relation to ampicillin, with resistance frequencies exceeding 85%. Elevated resistance rates were also documented for ciprofloxacin, ceftazidime, and cefoxitin, registering above the 65% threshold. *S. aureus* demonstrated resistance rates surpassing 50.0% for agents such as penicillin G, streptomycin, erythromycin, and azithromycin. However, susceptibility remained relatively high with resistance rates falling below 30.0% for vancomycin, gentamicin, and tobramycin. Resistance rates for acetazolamide and teicoplanin were conspicuously elevated. For comprehensive data and a breakdown of these observations, refer Tables 2 and 3.

**Table 2:** Analysis of the drug resistance of major Gram-negative bacteria to commonly used antibiotics

Drugs	*K. pneumoniae* (*n* = 19)		*E. coli* (*n* = 16)		*P. aeruginosa* (*n* = 15)	
	Resistant strains (*n*)	Drug resistance rate (%)	Resistant strains (*n*)	Drug resistance rate (%)	Resistant strains (*n*)	Drug resistance rate (%)
Clindamycin	4	21.05	3	18.75	3	20.00
Ampicillin	17	89.47	14	87.50	14	93.33
Streptomycin	11	57.89	8	50.00	8	53.33
Cefoxitin	13	68.42	11	68.75	9	66.67
Ceftazidime	15	78.95	12	75.00	12	80.00
Cefepime	7	36.84	6	37.50	10	66.67
Ciprofloxacin	15	78.95	12	75.00	14	93.33
Levofloxacin	9	47.37	6	37.50	7	46.67
Norfloxacin	9	47.37	8	50.00	5	33.33
Gatifloxacin	6	31.58	6	37.50	0	0.00
Amikacin	2	10.53	1	6.25	0	0.00
Meropenem	0	0.00	0	0.00	0	0.00
Imipenem	0	0.00	0	0.00	0	0.00
Teicoplanin	0	0.00	0	0.00	0	0.00

**Table 3:** Analysis of the drug resistance of major Gram-negative bacteria to commonly used antibiotics

Drugs	*S. aureus* (*n* = 14)	
	Resistant strains (*n*)	Resistant rate (%)
Penicillin G	13	92.86
Streptomycin	10	71.43
Erythromycin	11	78.57
Azithromycin	7	50.00
Vancomycin	2	14.29
Gentamicin	4	28.57
Tobramycin	2	14.29
Amoxicillin	7	50.00
Acetazolamide	0	0.00
Teicoplanin	0	0.00

### 3.3 Univariate risk assessment for pulmonary infections in elderly cardiovascular patients

No significant difference was noted in the incidence of pulmonary infection in elderly patients with cardiovascular disease of different genders (*P* > 0.05). The incidence of pulmonary infection was greater in elderly patients with cardiovascular disease of age ≥70 years, history of diabetes, invasive operation, Ⅲ–Ⅳ grade cardiac function, hypoproteinemia, and hospital stay ≥10 days than in patients of age <70 years, having no history of diabetes, no invasive operation, I–II grade cardiac function, no hypoproteinemia, and hospital stay <10 days (*P* < 0.05) (Table 4).

**Table 4:** Univariate analysis of pulmonary infection in elderly patients with cardiovascular disease

General information		*n*	Infected group (*n* = 71)	Uninfected group (*n* = 529)	*χ* ^2^ value	*P* value
Gender (cases)	Male	312	38	274	0.075	0.785
	Female	288	33	255		
Age (years)	＜70	237	19	218	5.469	0.019
	≥70	363	52	311		
History of diabetes	None	542	57	485	9.318	0.002
	Yes	58	14	44		
Invasive operation	None	428	41	387	7.270	0.007
	Yes	172	30	142		
Cardiac function grade	I–II grade	319	29	290	4.910	0.027
	Ⅲ–Ⅳ grade	281	42	239		
Hypoproteinemia	None	434	43	391	5.575	0.018
	Yes	166	28	138		
Hospital stay (days)	＜10	491	49	442	8.902	0.003
	≥10	109	22	87		

### 3.4 Multivariate logistic regression analysis for predicting pulmonary infections in elderly patients with cardiovascular disease

A multivariate logistic regression model was built using simply the components that showed statistically significant differences. The assignment of values was as follows: the incidence of pulmonary infection served as the dependent variable, while variables exhibiting statistical significance functioned as independent variables. These independent variables included: age (<70 years = 0, ≥70 years = 1), invasive operation (none = 0, yes = 1), history of diabetes (none = 0, yes = 1), cardiac function grade (I–II = 0, III–IV = 1), hypoproteinemia (none = 0, yes = 1), and duration of bed rest (<10 days = 0, ≥10 days = 1). According to the logistic regression output (Table 5), factors such as advanced age, history of diabetes, hypoproteinemia, invasive operation, high cardiac function grade, and hospital stay ≥10 days were independent risk factors for pulmonary infection in elderly patients with cardiovascular disease.

**Table 5:** Logistic multivariate regression analysis of pulmonary infection in elderly patients with cardiovascular disease

Observation index	*β*	SE	Wald	*P*	Exp (*B*)	95% CI
Age	1.045	0.343	9.262	0.002	2.844	1.451–5.574
History of diabetes	1.586	0.392	16.382	<0.001	4.884	2.266–10.527
Invasive operation	1.249	0.332	14.124	<0.001	3.488	1.818–6.692
Cardiac function grade	2.015	0.355	32.192	<0.001	7.499	3.739–15.040
Hypoproteinemia	1.293	0.311	17.235	<0.001	3.642	1.978–6.705
Hospitalization time	1.520	0.337	20.321	<0.001	4.573	2.361–8.857
Constant	−5.166	0.500	—	—	—	—

### 3.5 Establishment and validation of logistic regression model for pulmonary infection risk

**Figure 1:**
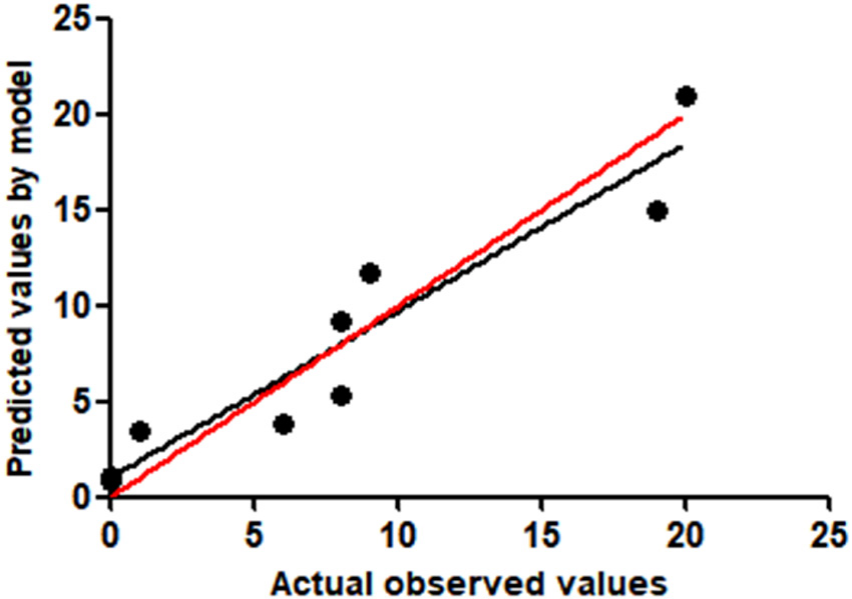
Hosmer–Lemeshow goodness-of-fit test to evaluate the calibration ability of the prediction model.

**Figure 2:**
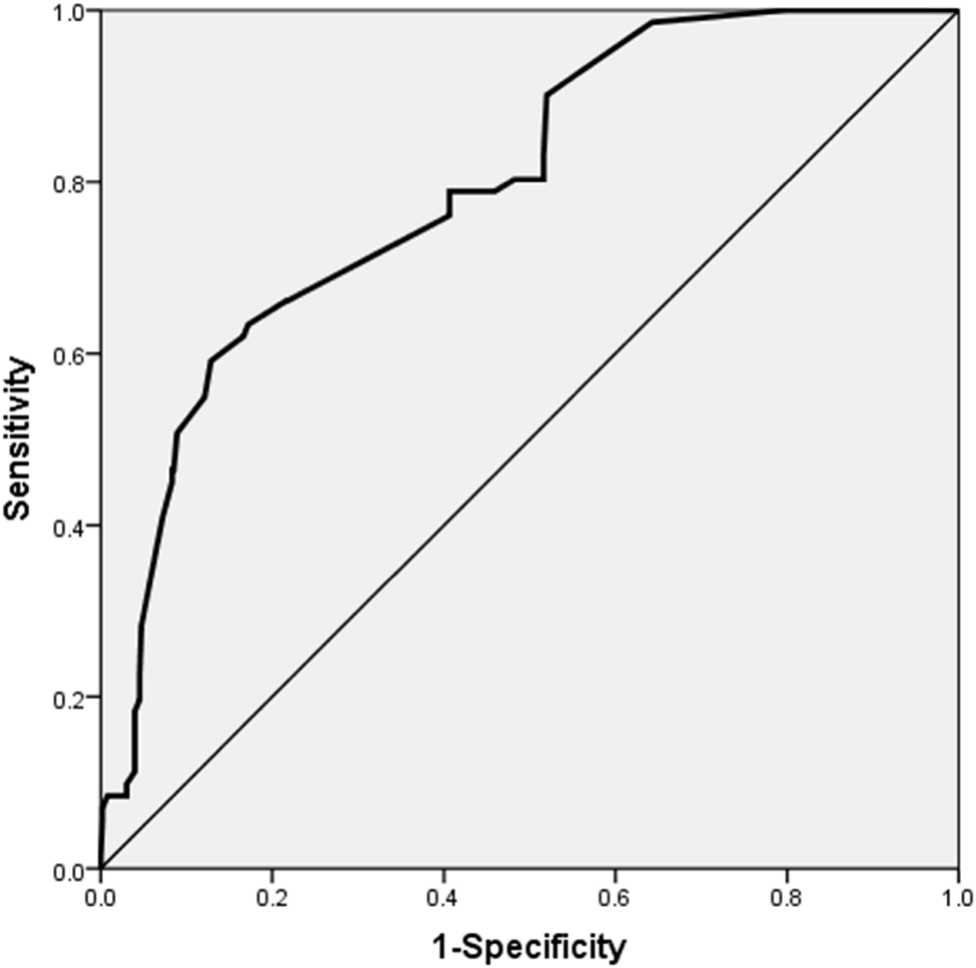
ROC curve was used to evaluate the risk scoring system.

The regression equation for the probability of pulmonary infection in elderly patients with cardiovascular diseases is as follows: *P* = 1/[1 + e^−(−5.166 + 1.045* Age + 1.586* Diabetes history + 1.249* Invasive operation + 2.015* Cardiac function grade + 1.293* Hypoalbuminemia + 1.520* Hospital stay)^]. The Hosmer–Lemeshow test was applied to evaluate the regression model, yielding a *P*-value of 0.236 (Figure 1), indicating a suitable fit. Internal validation of the model revealed an area under the ROC curve (AUC) of 0.795 [95% CI (0.743–0.847), *P* < 0.001], denoting high discriminative power. The model demonstrated a sensitivity of 63.40% and a specificity of 82.80%. The optimal cut-off point was identified as 0.13, as determined by the maximum Youden index (Figure 2).

## 4 Discussion

In geriatric patients afflicted with cardiovascular diseases, notably those with coronary artery disease, the myocardial tissue frequently exists in a prolonged state of hypoxia and ischemia. This condition contributes to compromised systolic heart function and pulmonary capillary congestion, thereby creating a conducive environment for the colonization of various pathogenic microorganisms, ultimately predisposing the patient to pulmonary infections. Additionally, in the context of heart failure, an elevation in pulmonary arterial pressure manifests, culminating in perturbations in the pulmonary circulation and consequently augmenting the susceptibility of the pulmonary system to infection. Moreover, cardiac enlargement during episodes of heart failure indirectly exerts compressive forces on posterior mediastinal structures, such as the trachea, thereby resulting in compromised ventilation and an elevated risk of respiratory infections [10,11].

In this study, nosocomial infections were more common among the elderly with cardiovascular disease (11.83%), according to a study by van Mens et al., than pulmonary infections among those with cardiovascular disease who had been hospitalized [12]. The older patients in this research may have a higher risk of lung infection because of their weakened immune systems, diminished resistance to disease, and impaired tissue and organ functioning as a result of the disease’s prolonged course. We isolated 93 positive strains from 71 patients in the infected group; the strains were mainly Gram-negative bacilli, accounting for 72.04%. Ranzani et al. [13] also found that the proportion of Gram-negative bacilli in hospital-acquired pneumonia has been increasing each year and are the main pathogenic bacteria responsible for hospital-acquired pneumonia; our study results are consistent with this finding. The reason for Gram-negative bacilli being the primary pathogen is attributed to this bacterium’s multiple drug resistance mechanisms, such as AmpC beta-lactamases and extended spectrum beta-lactamase enzymes; multidrug resistance occurs and is also related to the irrational use of antibacterial drugs in clinical practice [14]. Among the pathogen types isolated in this study, Gram-positive bacteria comprised mainly of *S. aureus*, which belongs to the group of symbiotic opportunistic pathogens that are common in parasitic sites within the population [15]. *K. pneumoniae*, *E. coli*, and *P. aeruginosa* were the most commonly found Gram-negative bacteria in this investigation. For example, *K. pneumoniae* is one of the major pathogens causing nosocomial infections and may cause a variety of illnesses affecting the gastrointestinal, respiratory, and urinary systems. The presence of *E. coli* is considered typical of gut flora. When the body’s immunity decreases, the likelihood of pathogenic bacteria colonizing the lungs or urinary tract increases, leading to nosocomial infections. In patients who have undergone invasive operations, *P. aeruginosa* is one of the significant pathogens leading to nosocomial infections [16].

The use of immunosuppressants and antibacterial drugs has become increasingly common in recent years, and the number of invasive surgeries and the reliance on antibacterial drugs have both contributed to the increase in the prevalence of drug-resistant strains of the most common infectious pathogens, such as *S. aureus*. The percentage of *S. aureus* strains resistant to gentamicin, vancomycin, and tobramycin was below 30.0%, whereas the percentage of strains sensitive to acetazolamide and teicoplanin was rather high. Among the 14 strains of *S. aureus* isolated in this study, there were no resistant strains to acetazolamide and teicoplanin. In this study, the most sensitive drugs for three Gram-negative bacteria, *K. pneumoniae*, *E*. *coli*, and *P*. *aeruginosa*, were imipenem, teicoplanin, and meropenem, with sensitivity rates of more than 90%, which could be used as the preferred antibacterial drugs when infected with this pathogen. An ampicillin resistance rate of over 90% was found, with ciprofloxacin, ceftazidime, and cefoxitin showing greater resistance rates in Gram-negative bacteria. Gram-negative bacteria in this research had a high resistance rate to antibiotics because they developed extended range β-lactamases. With the wide application of third-generation cephalosporins in lower respiratory tract infection, the emergence of third-generation cephalosporin-resistant bacteria has been observed. The main mechanism of bacterial drug resistance is the production of specific β-lactamase. The super-broad-spectrum β-lactamase bacteria are multi-drug resistant bacteria, which are easy to cause explosive epidemics and difficult to control. Under normal circumstances, the production of cephalosporin enzyme is low, but a large number of β-lactamase antibiotics induce the production of cephalosporin enzyme, making the strain resistant to cephalosporins and penicillin antibiotics, and highly sensitive to carbapenems, aminoglycosides, and enzyme-inhibiting antibiotics, Therefore, carbapenems, aminoglycosides, and enzyme inhibitor antimicrobials should be considered for clinical treatment of ultra-broad spectrum β-lactamase bacteria [17,18].

In this study, in the elderly with cardiovascular disease, the risk of lung infection was 2.844 times greater in patients aged ≥70 years compared to patients aged <70 years. With the increase in age, the body’s defense and immunity decrease; elderly patients are often affected with various underlying diseases and need to take broad-spectrum antibiotics intermittently for a long time, thus becoming a high-risk group for pulmonary infection. Mairuhu et al. [19] found that diabetes is one of the risk factors for nosocomial pneumonia; in fact, in this study, we observed diabetes as a predisposing factor for pulmonary infection in elderly patients with cardiovascular disease, consistent with the above research results. In patients with diabetes, the plasma hypertonic environment inhibits the chemotaxis and phagocytosis of neutrophils and inhibits T lymphocytes, leading to a decrease in immune function. In addition, the high-glucose environment is also more conducive to bacterial growth and reproduction, so special attention should be paid to blood glucose control in hospitalized elderly patients with cardiovascular disease [20]. Serum albumin is one of the important agents involved in the maintenance of the immune mechanism, and it also reflects the nutritional status of patients. In this study, patients with hypoalbuminemia are prone to pulmonary infection; such patients are at increased risk of pulmonary infection due to decreased immunity caused by malnutrition. During invasive operations such as nasal feeding, catheterization, and tracheal intubation, either incomplete disinfection or local mucosal damage leads to an increased risk of nosocomial infection [21]. In this study, patients who had undergone invasive surgeries had a greater chance of developing a lung infection than those who had not. Invasive procedures weaken the body’s immune system and make it easier for pathogens to invade. Under mechanical ventilation, the patient’s airway is continuously opened and exposed to the outside world; outside air cannot be filtered, and secretions accumulate between the glottis and the tracheal tube balloon. Pathogens can easily invade the lower respiratory tract and increase the risk of pulmonary infection [22]. Ericson et al. [23] indicated a higher risk of pulmonary infection in patients with coronary heart disease who had higher cardiac function grades. This study also shows that cardiac function grades are an independent risk factor for postoperative infection. Cardiac function grade often represents the degree of vascular lesions in patients. Myocardial ischemia induces chemokines to activate the adherence of leukocytes to necrotic tissues and cells and their removal. However, this process releases a large number of reactive oxygen species and free radicals, which can easily lead to inflammatory reactions, aggravate injury, and induce infection [24]. Concurrently, patients with longer hospital stays are at increased risk of infection.

To better detect and manage the risk of lung infection in patients, this research also built a risk prediction model by screening risk variables in older patients with cardiovascular disease. The ROC curve, with sensitivity on the ordinate and 1-specificity on the abscissa, was used to assess the reliability of the model. Generally, an AUC > 0.7 indicates that the model has a high accuracy; the greater the AUC, the higher the accuracy [25]. The risk model established in this study exhibited good discriminant validity and can thus be applied to clinical diagnosis and treatment. Medical staff can establish risk levels for patients according to the model and perform targeted interventions to improve the utilization efficiency of medical resources. Although the risk prediction model for pulmonary infection in elderly patients with cardiovascular disease was established, there are still many shortcomings. For example, the data source in this study is relatively limited; therefore, multi-center and large-sample data are required for subsequent validation of the model. Furthermore, the indicators included in this study are daily management indicators of nosocomial infection, and relevant iatrogenic indicators were not included; therefore, the model needs further improvement.

To conclude, this study analyzed the clinical data of elderly patients with cardiovascular disease, screened risk factors based on the regression model, and constructed a preliminary risk score prediction model for pulmonary infection based on the regression model. In older patients with cardiovascular conditions in the hospital, the model has strong discriminant validity and may be utilized to provide a foundation for the early identification of high-risk groups and effective prevention and management of pulmonary infection. The limitation of this study is that the selected cases are all cases in our hospital, which may have geographical limitations, so the selection range of cases can be expanded for further exploration, and further prospective studies are needed to verify the effectiveness and applicability of this prediction model. The clinical significance of this study is to identify elderly patients with cardiovascular disease who are susceptible to lung infection through the risk prediction model, so as to prevent and control it as early as possible.
